# ODYSSEY-HCM trial (mavacamten in symptomatic nonobstructive hypertrophic cardiomyopathy)

**DOI:** 10.1093/ehjcvp/pvaf077

**Published:** 2025-10-14

**Authors:** Emanuele Monda, Giuseppe Limongelli

**Affiliations:** Department of Translational Medical Sciences, Inherited and Rare Cardiovascular Diseases, University of Campania ‘Luigi Vanvitelli’, Via L. Bianchi 1 c/o Monaldi Hospital, Naples 80131, Italy; Department of Translational Medical Sciences, Inherited and Rare Cardiovascular Diseases, University of Campania ‘Luigi Vanvitelli’, Via L. Bianchi 1 c/o Monaldi Hospital, Naples 80131, Italy

Hypertrophic cardiomyopathy (HCM) is a myocardial disease characterized by increase left ventricular (LV) wall thickness not solely explained by abnormal loading conditions.^1^ According to the presence of LV outflow tract obstruction (LVOTO), HCM can be classified as obstructive or non-obstructive.^1^ In patients with obstructive HCM, mavacamten—a selective, allosteric, reversible, small-molecule cardiac myosin inhibitor—has been shown to reduce myosin ATPase activity, as well as the increased contractile force and calcium sensitivity induced by sarcomeric variants.^2^ In clinical trials enrolling patients with obstructive HCM, mavacamten reduced LVOTO, and improved exercise capacity, symptoms and patient-reported health status compared with placebo.^3^ Based on the rationale that mavacamten targets the core molecular defect of HCM, it has been hypothesized that the drug might also provide beneficial effects in non-obstructive HCM. In a multicentre, double-blind, placebo-controlled phase II trial, treatment with mavacamten was significantly associated with improvements in biomarkers [i.e. reduction from baseline to week 16 in N-terminal pro-B-type natriuretic peptide (NT-proBNP) and troponin I], compared with placebo.^4^ The preliminary findings supported the development of a phase III trial to further evaluate the potential role of mavacamten in patients with non-obstructive HCM.

The ODYSSEY-HCM trial investigated whether mavacamten improves functional capacity and patient-reported health status in patients with symptomatic non-obstructive HCM.^5^ The study enrolled adult patients with New York Heart Association (NYHA) class II or III symptoms, a peak LVOT gradient of less than 30 mmHg at rest or 50 mmHg with provocation, a Kansas City Cardiomyopathy Questionnaire clinical summary score (KCCQ-CSS) of 85 or lower, a LV ejection fraction (LVEF) of at least 60%, and elevated NT-proBNP levels. The trial included 580 patients with a mean age of 56 ± 15 years (54% male) and a mean time from the initial HCM diagnosis of 10.3 ± 9.3 years. From baseline to week 48, the two primary endpoints—change from baseline in peak oxygen uptake and in the KCCQ-CSS—did not differ significantly between the mavacamten and the placebo group. In contrast, a high rate of adverse events was observed among patients treated with mavacamten. Specifically, a reduction in LVEF to below 50% occurred in 21.5% of patients receiving mavacamten compared with 1.7% of those receiving placebo.

The results of the ODYSSEY-HCM trial support the concept that non-obstructive HCM may have distinct mechanisms underlying symptom burden compared with obstructive HCM. In these patients, where outflow obstruction is absent, symptoms and exercise limitations are more likely attributable to subendocardial ischaemia, diastolic dysfunction, reduced ventricular compliance, and abnormal cellular energetics. On the other hand, even without reaching statistical significance, the results of the ODYSSEY-HCM trial showed improvements with mavacamten compared to placebo. At 48 weeks, peak oxygen uptake increased by 0.52 mL/kg/min with mavacamten vs. 0.05 mL/kg/min with placebo (between-group difference: 0.47 mL/kg/min; 95% CI: −0.03 to 0.98; *P* = 0.07). Similarly, the KCCQ-CSS improved by 13.1 points with mavacamten vs. 10.4 points with placebo (between-group difference: 2.7 points; 95% CI: −0.1 to 5.6; *P* = 0.06). Future studies with larger populations, longer follow-up, and refined patient selection criteria will be essential to clarify the role of myosin inhibition in non-obstructive HCM. At the same time, research should also aim to identify novel therapeutic targets to broaden the treatment landscape for patients with non-obstructive HCM.

## Data Availability

The data underlying this article were taken from the quoted references.
